# The Pathophysiology of Sex Differences in Stroke Risk and Prevention in Atrial Fibrillation: A Comprehensive Review

**DOI:** 10.3390/medicina61040649

**Published:** 2025-04-01

**Authors:** Ibrahim Antoun, Georgia R. Layton, Ahmed Abdelrazik, Mahmoud Eldesouky, Mustafa Zakkar, Riyaz Somani, André Ng

**Affiliations:** 1 Department of Cardiology, University Hospitals of Leicester NHS Trust, Glenfield Hospital, Leicester LE3 9QP, UK; ia277@leicester.ac.uk (I.A.); ahmed.abdelrazik@leicester.ac.uk (A.A.); mie7@leicester.ac.uk (M.E.); riyaz.somani@uhl-tr.nhs.uk (R.S.); 2 Department of Cardiovascular Sciences, Clinical Science Wing, University of Leicester, Glenfield Hospital, Leicester LE3 9QP, UK; grl13@leicester.ac.uk (G.R.L.); mustafazakkar@me.com (M.Z.); 3 Department of Cardiac Surgery, University Hospitals of Leicester NHS Trust, Glenfield Hospital, Leicester LE3 9QP, UK; 4 National Institute for Health Research, Leicester Research Biomedical Centre, Leicester LE3 9QP, UK; 5 Leicester British Heart Foundation Centre of Research Excellence, Glenfield Hospital, Leicester LE3 9QP, UK

**Keywords:** atrial fibrillation, stroke, gender disparity, gender gap, pathophysiology

## Abstract

Atrial fibrillation (AF) is the most common chronic arrhythmia and is a leading cause of stroke, with well-documented differences in pathophysiology, clinical manifestations, and prognosis according to the sex of the patient. This review provides an overview of known or hypothesized sex differences in physiology and stroke risk for patients with AF. Women are reported to have more extensive fibrosis of the left atrium, different functional properties of the atria, and higher sensitivity to prothrombotic stimuli, especially after menopause. Variations in stroke risk with AF are linked to age, hypertension, diabetes, and chronic kidney disease; overall, women have worse outcomes. The widely clinically implemented CHA_2_DS_2_-VASc score no longer considers sex as a variable, and its propriety for women is still debated. However, women are usually under prescribed anticoagulation despite having a higher long-term risk of stroke compared to men, suggesting a lack of equity of treatment for certain patient groups. New AI-based risk stratification models and precision medicine approaches are potentially useful in reducing these gaps. Future work should also aim to improve sex-based predictive models, considering different gender categories, and understanding the part played by hormonal alterations, atrial structural alterations, and thromboembolic risk in the treatment of AF.

## 1. Introduction

Atrial fibrillation (AF) is the most common sustained arrhythmia worldwide, posing significant public health and clinical challenges due to its associated risks of stroke, heart failure, and mortality [1,2]. AF management can be challenging, especially in developing countries with limited resources [3,4,5,6,7,8,9,10]. The underlying mechanisms of AF are highly complex and multifactorial, involving structural, genetic, and electrophysiological changes in the atria. Among these factors, hormonal influences have emerged as critical contributors to the pathophysiology and incidence of AF and resultant AF-related strokes, particularly regarding sex-specific differences. Stroke is a major complication of AF. AF is the most common cause of cardio-embolic strokes, and there is an enhanced mortality profile of strokes resulting from AF compared to strokes from other causes [11].

Women with AF experience more severe arrhythmia-related symptoms and have a higher risk of stroke than men [12], even though men have a greater lifetime risk of AF [13]. These disparities are partly attributable to structural and electrophysiological differences, including increased atrial fibrosis in women and larger atrial sizes in men. Furthermore, the susceptibility to and progression of AF are both influenced by hormonal fluctuations, with particular importance attached to menopause. Many existing risk stratification and treatment strategies fail to consider these sex-specific factors. Until 2024, the CHA_2_DS_2_-VA score considered female sex as a risk factor [14]. However, its predictive value is debated [14], with evidence suggesting that even without the inclusion of biological sex, its ability to discriminate thromboembolic risk in patients with AF remains good [15]. The recommendation from the 2024 European guideline updates followed an agreement that female sex is an age-dependent stroke risk modifier rather than an independent biological risk factor. It was also deemed to be exclusionary based on sex assigned at birth. It introduced clinical complexity for non-binary and transgender patients, as well as those receiving hormone replacement therapy (HRT). Anticoagulation and rhythm control strategies need to be individualized, regardless of biological sex, to optimize outcomes. AI and machine learning (ML) present promising personalized risk assessment and treatment [16] opportunities. AI-driven imaging and predictive modelling can improve AF detection and management, incorporating sex-specific data for targeted interventions.

This review examines the sex-specific mechanisms of AF and its associated stroke risk, with particular focus on the underlying pathophysiology in women compared to men. We reviewed the structural and electrophysiological differences, hormonal influences, and current risk stratification and anticoagulation therapy challenges. We also discussed potential solutions, such as artificial intelligence (AI)-driven personalized risk prediction and updated guideline recommendations.

## 2. Gender Disparity in the Mechanism of Thrombosis in Atrial Fibrillation

As discussed throughout this review, the incidences and prevalences of specific risk factors associated with AF and stroke development vary between the biological sexes, resulting in varied onsets of both conditions in particular circumstances unique to either males or females. Similarly, some mechanisms known to propagate AF or stroke risk are shared between the sexes.

### 2.1. Left Atrial Dysfunction

LA dysfunction plays a central role in AF-related stroke risk. Enlargement of the LA, driven by conditions such as hypertension, heart failure, and valvular disease, leads to electrical and structural remodeling, increasing susceptibility to AF. This increased susceptibility to AF and the absence of proper physiological contraction results in blood pooling, the loss of laminar flow, and promotion of thrombus formation.

One example of electrophysiology disruption that can occur is LA low-voltage areas (LVAs). These regions of the LA demonstrate reduced electrical voltage during sinus rhythm and indicate degeneration of the atrial myocardium, often due to fibrosis, scarring, or structural remodeling. These areas are known to contribute to both the persistence of AF and its recurrence after ablation therapy. An observational study suggested that LVAs may be more prevalent in women than men. However, these areas generally become more frequent in both sexes with aging, larger atrial size, and the presence of comorbidities such as diabetes [17].

Sex variation in LA diameter, enlargement, and remodeling is difficult to quantify. However, we know that a greater LA diameter, or greater enlargement from the starting LA diameter, enhances the risk of stroke associated with AF [18]. Men typically have larger absolute LA diameters, but women tend to experience greater relative enlargement from their baseline LA size.

However, gender differences are known to exist within the relationship between LA diameter and other cardiovascular risk factors at a genetic level. In females, genetic risk for obesity appears to be a primary driver of increased diameter [19]. In contrast, in males, the presence of concomitant coronary artery disease or AF plays a more significant role. This highlights the multifactorial nature of LA remodeling and also suggests that certain traditional risk factors for AF, such as obesity, have a greater impact on female patients.

### 2.2. Prothrombotic State and Sex-Specific Influences

AF is known to facilitate a prothrombotic state by disrupting normal physiology across all domains of Virchow’s triad: stasis, endothelial injury, and hypercoagulability.

#### 2.2.1. Stasis

Inefficiency in the systolic function of the atria in AF leads to a decrease in stroke volume and an increase in stasis, particularly in patients for whom the LA kick is a major contributor to stroke volume. Furthermore, the LA appendage, which is normally regarded as a nonfunctional structure, is very muscular in its structure. When normal atrial contraction and flow dynamics are lost, the elongated shape of the appendix, with a narrow orifice, also promotes blood stasis. It thus becomes a primary site for thrombus formation [20].

#### 2.2.2. Endothelial Injury and Function

Endothelial function plays a key role in regulating thrombosis. Flow-mediated shear stress regulates nitric oxide (NO) homeostasis, and its release decreases as flow velocity decreases [21]. NO is anti-thrombotic by inhibiting platelet aggregation, and animal studies have demonstrated reduced NO release in the context of impaired LA contraction [22]. Estrogen is known to enhance the production of endothelial NO synthase, a key regulator of NO expression, so estrogen is associated with enhanced NO bioavailability in females [23]. The loss of estrogen enhances oxidative stress, leading to the loss of NO bioavailability, and results in vascular endothelial dysfunction and injury [24]. This is believed to be a major contributor to the enhanced cardiovascular risk in women following menopause [25]. Endothelial injury and dysfunction also occur in the context of vascular inflammatory states. Driven by extracellular matrix turnover and the renin–angiotensin system, inflammation results in endothelial damage and, consequently, thrombogenesis through platelet activation. Women often exhibit higher levels of inflammatory markers released in this inflammatory process, such as CRP, suggesting that they may experience higher levels of inflammation and, therefore, may exhibit an enhanced prothrombotic state in AF compared to men [26]. However, this hypothesis remains disproven [27], as other key acute-phase inflammatory proteins, such as IL-6, tend to be expressed at lower levels in women than in men, contradicting this theory.

#### 2.2.3. Hypercoagulability

The loss of estrogen after menopause results in loss of the natural anticoagulant impact of estrogen. The estrogen paradox is that endogenous estrogen improves the cardiovascular risk profile, including the risk of thrombosis, in pre-menopausal women. When this protective effect is lost, the risk of thrombosis increases. However, the provision of exogenous estrogen to women at any time, generally as a contraceptive pre-menopause or as HRT post-menopause, results in a hypercoagulable state and enhances the risk of thrombosis [28]. This paradox results from variations in how endogenous and exogenous estrogen are metabolized [29]. In pre-menopausal women, natural estrogen helps to keep blood clotting in check by balancing procoagulant and anticoagulant factors. It boosts fibrinolysis by increasing tissue plasminogen activator, which helps to break down clots and suppresses thrombin formation to prevent excessive clotting. However, HRT can tip this balance in the opposite direction by raising levels of clot-promoting factors, such as Factor VII, Factor VIII, fibrinogen, and von Willebrand factor, while reducing natural anticoagulants, such as protein C, protein S, and antithrombin. It also makes it harder for the body to break down clots by increasing plasminogen activator inhibitor-1 (PAI-1). These effects are especially strong with oral estrogen, as it undergoes first-pass metabolism in the liver, amplifying its impact on clotting.

## 3. Gender Disparity in Atrial Myopathy

The structural and functional differences in atrial tissue between women and men greatly influence the risk of atrial myopathy and the development of AF. These differences are important in forming prevention and treatment strategies. Sex differences in atrial structure are seen, especially in the setting of LA size and function. A higher burden of atrial fibrosis has been found in women compared to men, which is a structurally based change known to be associated with AF [30,31]. This increased fibrosis in women may be linked to hormonal influences, as menopause-related changes can exacerbate the risk of AF in women, thereby attenuating the sex differences typically observed in AF prevalence [32,33]. Estrogen has been found to exert anti-fibrotic effects by inhibiting fibroblast proliferation and collagen deposition in the atrial myocardium. Experimental studies suggest that estrogen receptor activation downregulates the expression of pro-fibrotic genes, such as transforming growth factor-beta (TGF-β) and matrix metalloproteinases (MMPs), thereby limiting structural remodeling of the left atrium [34,35]. Post-menopausal estrogen decline leads to increased atrial fibrosis, contributing to the greater burden of atrial scarring observed in women.

Furthermore, the left atrial diameter (LAD) is often larger in men, with defined thresholds indicating dilated atria set differently for men and women [31,36]. However, the extent of LA enlargement does not differ significantly between genders, suggesting that although size may vary, the underlying risk factors for AF may be similarly impactful across the sexes [31]. Functional differences in atrial tissue are also notable. Research has shown that women exhibit different patterns of atrial function, including variations in atrial strain and deformation, which are critical for effective atrial contraction and relaxation [37,38]. A cardiovascular magnetic resonance imaging (CMR) study found that age and sex significantly affect atrial volumes and phasic function, indicating that these factors must be considered in evaluating atrial health [39].

**Figure 1:**
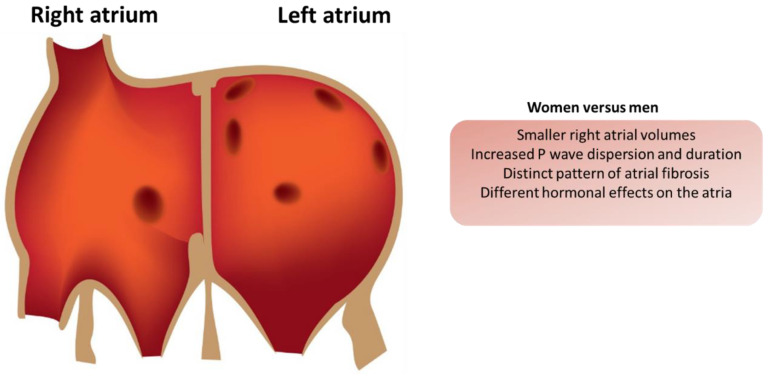
Electrophysiological differences in the atria of men and women.

Furthermore, epicardial adipose tissue (EAT) has been reported to stimulate inflammation and structural alteration of the atria, with different effects between men and women [40]. This indicates that the metabolic environment of the atria may influence the observed sex differences in AF risk. Electrophysiological differences also exist, further complicating the scenario of AF risk between the genders (Figure 1). It has been observed that women have relatively slower conduction in the atria, which may make them more prone to arrhythmias [41]. These electrophysiological properties are known to be modulated by the hormonal milieu, particularly sex steroid hormones, which may make women more prone to AF [33,42]. In addition, the interaction between structural remodeling and electric activity is important since LA dysfunction is a more sensitive marker of AF recurrence than LA size alone [43].

The presence of atrial fibrosis affects the mechanical and electrical properties of the atria, which can lead to ineffective atrial contraction and the stasis of blood flow. In men, the larger atrial size may contribute to a greater overall risk of thrombus formation; however, the fibrotic changes observed in women can result in a more pronounced risk of stroke due to reduced contractility and the increased likelihood of blood pooling in the LA appendage (LAA) [37,38]. This is particularly relevant for women with persistent AF, where structural remodeling is often more extensive compared to their male counterparts [39].

The influence of genetic and hormonal factors on atrial myopathy and the associated risk of stroke constitutes a multifaceted area of research that highlights the interplay between biological predispositions and environmental elements. Hormonal influences, particularly sex hormones, contribute to the risk of atrial myopathy and stroke.

## 4. The Role of Pregnancy and Hormones on AF Risk

Various factors, including hormonal changes during pregnancy and the effects of hormonal treatments in males and females, influence the pathophysiology of AF. Understanding these influences is crucial for managing AF effectively, particularly in women who are pregnant or undergoing hormonal therapies. Pregnancy induces significant physiological changes that can precipitate AF. Hemodynamic alterations, such as increased blood volume and cardiac output, may exacerbate underlying cardiac conditions, especially in women with pre-existing heart disease, such as mitral valve disease [44]. These changes can lead to new-onset AF, frequently observed in pregnant women, particularly those with congenital or rheumatic heart disease [45]. The heightened cardiovascular demands during pregnancy can unmask latent arrhythmias, making AF a notable concern [46].

**Figure 2:**
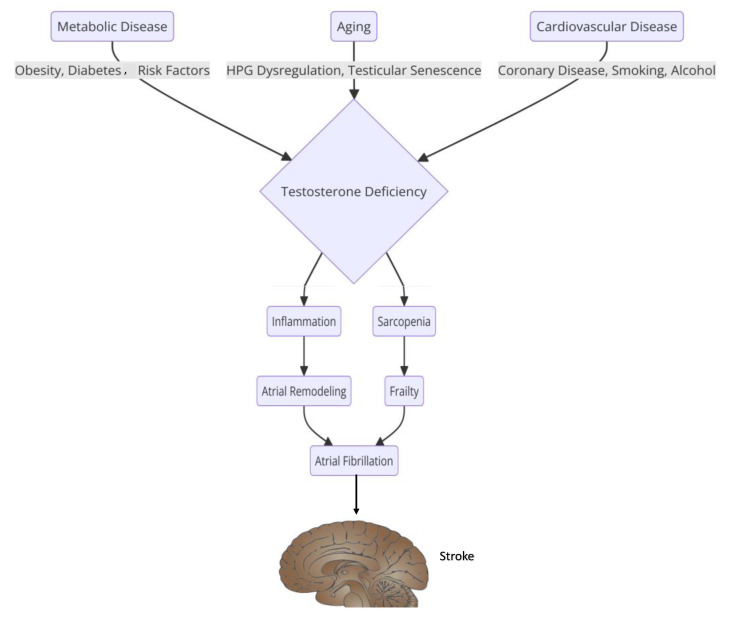
The relationship between sex hormones and the pathophysiology of atrial fibrillation.

Furthermore, the management of AF in pregnant women often involves the use of beta-blockers and calcium-channel blockers, which are preferred for rate control due to their safety profile [47,48]. Hormonal treatments, particularly those involving estrogen and progesterone, can also play a role in the pathophysiology of AF. Estrogen has been shown to have both protective and proarrhythmic effects on the heart. Some studies suggest that estrogen may enhance the risk of AF by promoting structural remodeling of the atria. In contrast, others indicate that it may have a protective effect against the development of AF in post-menopausal women [49,50]. The interplay between hormonal treatments and AF is complex, as hormonal fluctuations can influence the autonomic nervous system, potentially leading to increased susceptibility to arrhythmias [51]. In males, hormonal influences on AF are less frequently discussed, but testosterone levels have been implicated in the pathophysiology of AF (Figure 2). Low testosterone levels have been associated with an increased risk of AF, suggesting that hormonal balance is crucial for maintaining normal cardiac rhythm [49]. Additionally, systemic inflammation, which can be influenced by hormonal status, has been linked to the development and persistence of AF [52].

## 5. Gender Disparities in Stroke Risk Factors in AF Patients

AF is a significant risk factor for ischemic stroke, with evidence consistently illustrating gender-based differences in stroke risk and clinical outcomes.

One of the primary contributors to the increased stroke risk in women with AF is age. Women tend to develop AF later in life than men, and as age is a strong independent risk factor for stroke. This delay in diagnosis and management may contribute to poorer outcomes, as proposed in two meta-analyses [53,54]. Hypertension, one of the most significant modifiable stroke risk factors, is more prevalent in women with AF, further exacerbating their thromboembolic risk [55]. Similarly, diabetes mellitus has been shown to have a greater relative impact on stroke risk in women compared to men, emphasizing the need for aggressive risk-factor modification in female AF patients [56]. Furthermore, diabetic women have a greater risk of recurrence than men >70 years old, supporting a high-risk “time window” in post-menopausal, elderly diabetic women [57].

Beyond age, hypertension, and diabetes, other stroke risk factors exhibit gender disparities in AF patients. Obesity, a well-established risk factor for both AF and stroke, affects men and women differently. Although obesity contributes to AF incidence in both sexes, women with obesity tend to have a more pronounced prothrombotic profile, potentially increasing their stroke risk more than men [58]. Dyslipidemia, another key vascular risk factor, is often under-recognized and undertreated in women, leading to suboptimal stroke prevention efforts [59]. Smoking and alcohol consumption, although traditionally more prevalent in men, have been shown to exert a disproportionately higher stroke risk in women with AF, possibly due to sex-based differences in vascular biology and inflammatory responses [60].

Another critical risk factor is chronic kidney disease (CKD), which has been identified as an independent predictor of stroke in AF patients. Women with AF and CKD face a higher stroke risk than men, which may be due to differences in renal function decline, endothelial dysfunction, and anticoagulation response [61]. Heart failure, frequently coexisting with AF, also demonstrates a gender-specific stroke risk. Although men with AF and heart failure more commonly present with reduced ejection fraction, women more regularly exhibit preserved ejection fraction, which is associated with increased left atrial dysfunction and heightened thromboembolic risk [61]. Table 1 summarizes the sex-specific risk factors in males and females.

## 6. Anticoagulation and Stroke Prevention: Do Current Risk Scores Adequately Address Sex Disparities?

Growing evidence suggests that current risk stratification tools, such as the CHA_2_DS_2_-VASc score, may not fully capture sex-specific differences in the thromboembolic risk and anticoagulation response. Women with AF tend to bear a higher risk of stroke compared to men, even after adjusting for traditional risk factors, such as hypertension and diabetes. The mechanisms underpinning this disparity include variations in LA function, atrial fibrosis, and systemic prothrombotic states in women with AF [12,62].

AF patients are one example of a population where women have historically been considered an independent stroke risk factor. The CHA_2_DS_2_-VASc score, currently used for assessing stroke risk, gives one point for female sex. However, this classification has also been debated because of the concern that sex alone might not be the appropriate right risk modifier for making anticoagulation decisions for younger women. Studies indicate that the excess stroke risk in women is more pronounced in older populations (>65 years), whereas in younger women (≤65 years) without other risk factors, the risk may be overestimated [53,63].

Despite evidence of higher stroke risk, studies show that women with AF are less likely to receive anticoagulation therapy than men, particularly in cases where CHA_2_DS_2_-VASc scores are two or higher [64]. The underuse of direct oral anticoagulants (DOACs) in women may arise from concerns about bleeding, a perceived lower stroke risk, or differences in healthcare-seeking behavior. Conversely, some studies have suggested that women face a greater risk of bleeding complications on certain anticoagulants, particularly vitamin K antagonists, necessitating careful weighing of bleeding risk versus stroke prevention [10]. Furthermore, another study demonstrated a higher risk of bleeding in females compared to males receiving DOACs [65].

Introducing DOACs has enhanced access to anticoagulation and safety, particularly for previously underserved women. DOACs have demonstrated non-inferiority to warfarin for both men and women [66]. However, sex-specific pharmacokinetic differences must be considered when prescribing these agents [67].

The CHA_2_DS_2_-VASc score should be used cautiously for women as its application is complex. Incorporating gender in the CHA_2_DS_2_-VASc score complicates clinical practice for healthcare providers and consumers. It also excludes patients who are not categorized as male or female, such as individuals who are non-binary, transgender, or receiving hormone therapy. Therefore, the CHA_2_DS_2_-VA score (excluding gender) has effectively been established in the recent European Society of Cardiology (ESC) guidelines [14]. The transition to CHA_2_DS_2_-VA and personalized anticoagulation strategies represents a step towards more equitable stroke prevention. Future studies should investigate novel biomarkers, sex-specific predictors, and AI-driven risk models to refine anticoagulation approaches for patients with AF.

## 7. Future Directions

Given the established gender disparities in atrial myopathy and AF, future research should focus on refining risk stratification, developing personalized treatment strategies, and understanding the underlying biological mechanisms that contribute to sex-based differences in atrial remodeling. Several key areas warrant further investigation. AI has the potential to revolutionize the study and management of atrial myopathy by enhancing diagnostic accuracy, risk stratification, and treatment optimization. AI-powered imaging techniques, including deep learning models, applied to cardiovascular magnetic resonance imaging (CMR) and echocardiography, can refine the detection and quantification of atrial fibrosis, strain, and deformation across sexes. ML algorithms could develop sex-specific predictive models by integrating multimodal data, such as imaging, genetics, and clinical factors. These models remain in their early phases of implementation, but there already exist examples of deep neural networks being trained to predict new-onset AF in patients without a history of AF through ECG review. This can also be combined with clinical history data to predict those at risk specifically of AF-related stroke, not just the arrythmia itself [68].

The application of AI in personalized medicine could enhance risk assessment by improving CHA_2_DS_2_-VA models and anticoagulation response prediction. Wearable technologies combined with AI algorithms offer new opportunities for remote monitoring, allowing for the early detection of atrial myopathy and AF in high-risk populations. Additionally, AI-driven genomic analysis can uncover sex-specific genetic markers, paving the way for targeted interventions.

Moreover, future studies should incorporate transgender and non-binary populations into AF research to evaluate the impact of gender-affirming hormone therapy on atrial health. AI can facilitate large-scale data analysis to assess cardiovascular implications and guide clinical decision-making for gender-diverse individuals. By integrating AI into atrial myopathy research, clinicians can optimize treatment approaches and reduce gender disparities in AF outcomes. Future studies should validate AI-driven models across diverse populations to ensure their clinical applicability and effectiveness.

## 8. Limitations

The present review offers a comprehensive description of the common proven or hypothesized mechanisms for sex differences in stroke risk and the requirements for anticoagulation in patients with atrial fibrillation. This study did not use a systematic approach to article selection due to the heterogeneity of study designs and reported outcomes. Articles have been incorporated based on author-determined relevance, which may lead to selection bias. In addition, the heterogeneity of the included studies, which have different designs, populations, and methodological procedures, makes any direct or statistical comparison non-viable. Most available studies report binary, biological sex categories (male and female) and do not incorporate information about transgender and non-binary people. However, emerging evidence suggests that risks related to AF in these patient groups do not directly replicate those of their corresponding biological sex and may demonstrate unique risk profiles [69]. Therefore, this is not addressed in this review.

Moreover, many studies comparing sex differences in stroke risk did not include all the possible confounders, such as healthcare availability and adherence to anticoagulation, which could explain the observed differences. This is because there has been a change in the clinical practice guidelines. For example, female sex was previously used on its own to predict stroke risk in the CHA_2_DS_2_-VASc score, and this has since been changed. However, there is a need to confirm these alterations. Finally, although AI-based predictive models are suggested as a possible means to enhance risk prediction, there is scarce evidence of their application in real clinical practice. Thus, future work should include systematic reviews, gender-diverse participants, and validation of the AI-based recommendations for sex-specific AF risk and management.

## 9. Conclusions

Many mechanisms and risks of cardioembolic stroke in patients with AF are shared by all patients, regardless of gender. However, subtle sex-specific differences remain, driven by variations in LA structure, electrophysiological remodeling, thrombotic risk, and hormonal influences that enhance the sex-specific risk profile for women. Women generally have a higher stroke risk despite lower lifetime AF prevalence, likely due to increased atrial fibrosis, heightened inflammatory responses, and post-menopausal endothelial dysfunction. However, many hypotheses purporting these risks remain uncertain and further work is required to unequivocally prove the female-specific mechanisms driving these differences in clinical outcomes. Recognizing these and narrowing the disparity in outcomes between men and women is essential and will support sex-specific risk stratification and optimizing stroke prevention strategies for all patients. Future research should focus on integrating currently available sex-specific data into predictive models and refining personalized treatment approaches for AF-related stroke prevention.

## Figures and Tables

**Table 1:** Sex-specific risk factors for atrial fibrillation in males and females.

Risk Factor	Prevalence in Women with AF	Prevalence in Men with AF
Age	Higher, develop AF later in life	Develop AF at a younger age
Hypertension	More common, stronger association with stroke	Lower prevalence, but still a significant risk factor
Diabetes Mellitus	Higher impact on stroke risk, especially post-menopausal	Strong risk factor, but less pronounced effect on stroke
Chronic Kidney Disease	Greater stroke risk compared to men with CKD	Lower stroke risk than women with CKD
Dyslipidemia	Often undertreated, increasing stroke risk	More commonly treated, reducing risk impact
Obesity	Associated with higher prothrombotic state	Less impact on prothrombotic state
Smoking	Lower prevalence, but greater relative impact	Higher prevalence, but lower relative impact on stroke
Alcohol Consumption	Lower consumption, but higher stroke risk per unit	Higher consumption, but lower relative stroke risk
Heart Failure	More often with preserved EF, linked to higher stroke risk	More often with reduced EF, linked to stroke risk
Left Atrial Fibrosis	Greater fibrosis burden, linked to increased stroke risk	Lower fibrosis burden compared to women
Atrial Size	Smaller absolute size, but greater relative enlargement	Larger atria, which increases stroke risk
Hormonal Influence	Loss of estrogen increases prothrombotic risk post-menopause	Testosterone influence on AF risk remains unclear

AF: atrial fibrillation. CKD: chronic kidney disease. EF: ejection fraction.

## Data Availability

No new data were created or analyzed in this study.
